# Supplementary material for: Developing a machine learning model to identify protein–protein interaction hotspots to facilitate drug discovery
Source: PeerJ. 2020 Dec 7;8:e10381. doi: 10.7717/peerj.10381 (PMC7727375; doi:10.7717/peerj.10381)
Supplement: Supplemental Information 1 — The individual training and testing metrics of each of algorithms developed and tested per pipeline, and during different ensembling techniques. [file peerj-08-10381-s001.docx]

**Supplementary Information for “Developing a Machine Learning Model to Identify Protein-Protein Interaction Hotspots to Facilitate Drug Discovery”**

Rohit Nandakumar^1^; Valentin Dinu, PhD^1^

^1^ Program of Biomedical Informatics, College of Health Solutions, Arizona State University, Tempe, Arizona, United States of America

Corresponding Author:

Rohit Nandakumar

Address: Biomedical Informatics - ASU

Mayo Clinic, SC. Johnson Research Bldg

13212 E Shea Boulevard

Scottsdale AZ 85259

Email Address: [rnandaku@asu.edu](mailto:rnandaku@asu.edu)

**Phase 1**

Train metrics for SMOTE, NO PCA

|  | Accuracy | Precision | Recall | F1 | AUROC | Precision-Recall | MCC | Kappa | Sensitivity | Specificity |
| --- | --- | --- | --- | --- | --- | --- | --- | --- | --- | --- |
| LR | 0.8398 | 0.6252 | 0.8126 | 0.7057 | 0.8304 | 0.5526 | 0.609 | 0.5985 | 0.8126 | 0.8482 |
| RF | 0.9524 | 0.8795 | 0.9254 | 0.9015 | 0.943 | 0.8314 | 0.871 | 0.8702 | 0.9254 | 0.9607 |
| GBC | 0.9154 | 0.775 | 0.9064 | 0.835 | 0.9123 | 0.7246 | 0.7835 | 0.7787 | 0.9064 | 0.9182 |
| KNN | 0.88 | 0.6979 | 0.8658 | 0.7725 | 0.9604 | 0.636 | 0.7 | 0.6922 | 0.8658 | 0.8843 |
| MLP | 0.9769 | 0.9342 | 0.9714 | 0.952 | 0.9971 | 0.9142 | 0.9375 | 0.9368 | 0.9714 | 0.9785 |
| Gaussian | 0.7583 | 0.4934 | 0.6902 | 0.5738 | 0.7962 | 0.413 | 0.4248 | 0.4123 | 0.6902 | 0.7794 |

Train metrics for NO SMOTE, NO PCA

|  | Accuracy | Precision | Recall | F1 | AUROC | Precision-Recall | MCC | Kappa | Sensitivity | Specificity |
| --- | --- | --- | --- | --- | --- | --- | --- | --- | --- | --- |
| LR | 0.8483 | 0.6789 | 0.6869 | 0.681 | 0.7924 | 0.5407 | 0.5831 | 0.5817 | 0.6869 | 0.8979 |
| RF | 0.9185 | 0.7876 | 0.8968 | 0.8385 | 0.911 | 0.7307 | 0.7874 | 0.7843 | 0.8968 | 0.9252 |
| GBC | 1 | 1 | 1 | 1 | 1 | 1 | 1 | 1 | 1 | 1 |
| KNN | 1 | 1 | 1 | 1 | 1 | 1 | 1 | 1 | 1 | 1 |
| MLP | 0.974 | 0.9639 | 0.9252 | 0.9434 | 0.9571 | 0.9091 | 0.9275 | 0.9266 | 0.9252 | 0.989 |
| Gaussian | 0.7511 | 0.4822 | 0.6862 | 0.5648 | 0.7287 | 0.4043 | 0.4119 | 0.3985 | 0.6862 | 0.7711 |

Train metrics for SMOTE, PCA

|  | Accuracy | Precision | Recall | F1 | AUROC | Precision-Recall | MCC | Kappa | Sensitivity | Specificity |
| --- | --- | --- | --- | --- | --- | --- | --- | --- | --- | --- |
| LR | 0.8485 | 0.6396 | 0.819 | 0.718 | 0.8383 | 0.5667 | 0.6257 | 0.6166 | 0.819 | 0.8576 |
| RF | 0.9051 | 0.7623 | 0.8714 | 0.8125 | 0.8934 | 0.6944 | 0.753 | 0.7494 | 0.8714 | 0.9154 |
| GBC | 0.987 | 0.9666 | 0.9791 | 0.9726 | 0.999 | 0.9513 | 0.9643 | 0.9641 | 0.9791 | 0.9894 |
| KNN | 0.8373 | 0.6182 | 0.8117 | 0.7014 | 0.8284 | 0.5461 | 0.6032 | 0.5924 | 0.8117 | 0.8451 |
| MLP | 0.8384 | 0.6349 | 0.7572 | 0.6892 | 0.9039 | 0.5382 | 0.5868 | 0.5815 | 0.7572 | 0.8634 |
| Gaussian | 0.7731 | 0.5193 | 0.5551 | 0.5346 | 0.6977 | 0.3926 | 0.3869 | 0.3852 | 0.5551 | 0.8402 |

Train metrics for NO SMOTE, PCA

|  | Accuracy | Precision | Recall | F1 | AUROC | Precision-Recall | MCC | Kappa | Sensitivity | Specificity |
| --- | --- | --- | --- | --- | --- | --- | --- | --- | --- | --- |
| LR | 0.7766 | 0.5333 | 0.5598 | 0.5426 | 0.7015 | 0.4036 | 0.3983 | 0.3959 | 0.5598 | 0.8433 |
| RF | 0.9411 | 0.8542 | 0.9053 | 0.8787 | 0.9287 | 0.7956 | 0.8407 | 0.8398 | 0.9053 | 0.9521 |
| GBC | 1 | 1 | 1 | 1 | 1 | 1 | 1 | 1 | 1 | 1 |
| KNN | 1 | 1 | 1 | 1 | 1 | 1 | 1 | 1 | 1 | 1 |
| MLP | 0.8489 | 0.7686 | 0.5167 | 0.6145 | 0.7339 | 0.5111 | 0.5434 | 0.5256 | 0.5167 | 0.9511 |
| Gaussian | 0.7831 | 0.5441 | 0.5134 | 0.5262 | 0.6898 | 0.3939 | 0.3877 | 0.3861 | 0.5134 | 0.8661 |

Test metrics for SMOTE, NO PCA

|  | Accuracy | Precision | Recall | F1 | AUROC | Precision-Recall | MCC | Kappa | Sensitivity | Specificity |
| --- | --- | --- | --- | --- | --- | --- | --- | --- | --- | --- |
| LR | 0.7404 | 0.4694 | 0.9583 | 0.6301 | 0.8167 | 0.4594 | 0.5346 | 0.4641 | 0.9583 | 0.675 |
| RF | 0.8269 | 0.625 | 0.625 | 0.625 | 0.7562 | 0.4772 | 0.5125 | 0.5125 | 0.625 | 0.8875 |
| GBC | 0.7788 | 0.5172 | 0.625 | 0.566 | 0.725 | 0.4098 | 0.4228 | 0.4194 | 0.625 | 0.825 |
| KNN | 0.75 | 0.4643 | 0.5417 | 0.5 | 0.6771 | 0.3573 | 0.3364 | 0.3346 | 0.5417 | 0.8125 |
| MLP | 0.8365 | 0.6129 | 0.7917 | 0.6909 | 0.8208 | 0.5333 | 0.591 | 0.5822 | 0.7917 | 0.85 |
| Gaussian | 0.7019 | 0.4054 | 0.625 | 0.4918 | 0.675 | 0.3399 | 0.308 | 0.2942 | 0.625 | 0.725 |

Test metrics for NO SMOTE, NO PCA

|  | Accuracy | Precision | Recall | F1 | AUROC | Precision-Recall | MCC | Kappa | Sensitivity | Specificity |
| --- | --- | --- | --- | --- | --- | --- | --- | --- | --- | --- |
| LR | 0.7692 | 0.5 | 0.7917 | 0.6129 | 0.7771 | 0.4439 | 0.4849 | 0.4602 | 0.7917 | 0.7625 |
| RF | 0.8077 | 0.5667 | 0.7083 | 0.6296 | 0.7729 | 0.4687 | 0.5076 | 0.5019 | 0.7083 | 0.8375 |
| GBC | 0.8269 | 0.6667 | 0.5 | 0.5714 | 0.7125 | 0.4487 | 0.4733 | 0.4658 | 0.5 | 0.925 |
| KNN | 0.7981 | 0.56 | 0.5833 | 0.5714 | 0.7229 | 0.4228 | 0.4396 | 0.4394 | 0.5833 | 0.8625 |
| MLP | 0.7885 | 0.5455 | 0.5 | 0.5217 | 0.6875 | 0.3881 | 0.3869 | 0.3863 | 0.5 | 0.875 |
| Gaussian | 0.6731 | 0.375 | 0.625 | 0.4688 | 0.6562 | 0.3209 | 0.2706 | 0.2534 | 0.625 | 0.6875 |

Test metrics for SMOTE, PCA

|  | Accuracy | Precision | Recall | F1 | AUROC | Precision-Recall | MCC | Kappa | Sensitivity | Specificity |
| --- | --- | --- | --- | --- | --- | --- | --- | --- | --- | --- |
| LR | 0.8462 | 0.625 | 0.8333 | 0.7143 | 0.8417 | 0.5593 | 0.6238 | 0.6119 | 0.8333 | 0.85 |
| RF | 0.7692 | 0.5 | 0.75 | 0.6 | 0.7625 | 0.4327 | 0.4649 | 0.4468 | 0.75 | 0.775 |
| GBC | 0.7692 | 0.5 | 0.7083 | 0.5862 | 0.7479 | 0.4215 | 0.4453 | 0.4327 | 0.7083 | 0.7875 |
| KNN | 0.75 | 0.4688 | 0.625 | 0.5357 | 0.7062 | 0.3795 | 0.3766 | 0.3694 | 0.625 | 0.7875 |
| MLP | 0.8269 | 0.5882 | 0.8333 | 0.6897 | 0.8292 | 0.5287 | 0.5913 | 0.5745 | 0.8333 | 0.825 |
| Gaussian | 0.8269 | 0.625 | 0.625 | 0.625 | 0.7562 | 0.4772 | 0.5125 | 0.5125 | 0.625 | 0.8875 |

Test metrics for NO SMOTE, PCA

|  | Accuracy | Precision | Recall | F1 | AUROC | Precision-Recall | MCC | Kappa | Sensitivity | Specificity |
| --- | --- | --- | --- | --- | --- | --- | --- | --- | --- | --- |
| LR | 0.7788 | 0.52 | 0.5417 | 0.5306 | 0.6958 | 0.3874 | 0.3862 | 0.386 | 0.5417 | 0.85 |
| RF | 0.7692 | 0.5 | 0.6667 | 0.5714 | 0.7333 | 0.4103 | 0.426 | 0.4179 | 0.6667 | 0.8 |
| GBC | 0.8269 | 0.6364 | 0.5833 | 0.6087 | 0.7417 | 0.4674 | 0.4986 | 0.4979 | 0.5833 | 0.9 |
| KNN | 0.7788 | 0.5185 | 0.5833 | 0.549 | 0.7104 | 0.3986 | 0.4044 | 0.4032 | 0.5833 | 0.8375 |
| MLP | 0.8269 | 0.6364 | 0.5833 | 0.6087 | 0.7417 | 0.4674 | 0.4986 | 0.4979 | 0.5833 | 0.9 |
| Gaussian | 0.7308 | 0.4 | 0.3333 | 0.3636 | 0.5917 | 0.2872 | 0.196 | 0.1947 | 0.3333 | 0.85 |

Train ensemble (stacked) metrics:

| **Train** |  |  |  |  |  |  |  |  |  |  |
| --- | --- | --- | --- | --- | --- | --- | --- | --- | --- | --- |
|  | Accuracy | Precision | Recall | F1 | AUROC | Precision-Recall | MCC | Kappa | Sensitivity | Specificity |
| LR | 0.8201 | 0.5879 | 0.8016 | 0.6778 | 0.8137 | 0.519 | 0.5706 | 0.5573 | 0.8016 | 0.8258 |
| RF | 0.8775 | 0.6916 | 0.8716 | 0.7706 | 0.8755 | 0.6332 | 0.6978 | 0.6887 | 0.8716 | 0.8794 |
| GBC | 0.9758 | 0.9332 | 0.9672 | 0.9497 | 0.9728 | 0.9105 | 0.9342 | 0.9338 | 0.9672 | 0.9785 |
| KNN | 0.8369 | 0.6204 | 0.7953 | 0.6966 | 0.8225 | 0.5416 | 0.5963 | 0.5874 | 0.7953 | 0.8496 |
| MLP | 0.7981 | 0.5568 | 0.7428 | 0.6348 | 0.779 | 0.4739 | 0.511 | 0.4998 | 0.7428 | 0.8152 |
| Gaussian | 0.5551 | 0.3093 | 0.7023 | 0.4278 | 0.606 | 0.2872 | 0.1821 | 0.149 | 0.7023 | 0.5097 |
| All | 0.9658 | 0.9006 | 0.9624 | 0.9301 | 0.9646 | 0.876 | 0.9087 | 0.9075 | 0.9624 | 0.9668 |

Train ensemble (voting) metrics

|  | Accuracy | Precision | Recall | F1 | AUROC | Precision-Recall | MCC | Kappa | Sensitivity | Specificity |
| --- | --- | --- | --- | --- | --- | --- | --- | --- | --- | --- |
| Voting | 0.8789 | 0.7129 | 0.8189 | 0.7615 | 0.8581 | 0.6266 | 0.6845 | 0.681 | 0.8189 | 0.8974 |
